# Bibliographical Notices

**Published:** 1858-10

**Authors:** 


### BIBLIOGRAPHICAL NOTICES.

*Elements of Inorganic Chemistry, including the Applications of the Science in the Arts.* By THOMAS GRAHAM, F. R. S. Edited by HENRY WATTS, B. A., F. C. S., and ROBERT BRIDGES, M. D. Philadelphia, Blanchard & Lea, 1858.

Fifteen years ago, the distinguished professor of chemistry in the University College, London, issued the first edition of his Chemistry. It was so well received, that in 1850 he gave the world a second edition, of which the heading "Part I" gave promise of a complete treatise on the science, the work as published being confined to the inorganic department. The organic Chemistry was never published, and now the author has given us a third edition, still confined to the inorganic portion of the science. His English editor, Mr. Watts, has zealously aided him, and has incorporated in the work a large quantity of very valuable matter.

As the book now stands, we do not think ourselves guilty of exaggeration when we say that we consider it the best introduction to the study of chemistry which the student can find in the English language. It is copious upon those parts of chemical philosophy which are so obscure to the beginner, the different systems of atomic arrangement and the theory of salts and acids. The article, in the supplement, on chemical notation and classification is especially worthy of commendation, as an admirably clear statement of the present views of chemists on the subject of the relations of both elements and compounds to one another, and for a satisfactory analysis of the three systems of atomic weight. It is not, however, that portion of the book on which we would recommend the tyro to make his first attack.

In chemical physics the book is quite full enough, and perhaps unnecessarily minute. It is a department which is gradually assuming a rank for itself not independent of chemistry proper, but to a certain extent apart from it. The technical application of light to the analysis of crude sugars is described at considerable length, but we were surprised to find that the author stopped at the comparatively crude apparatus of Mitscherlich, without describing the far more elegant and delicate process of Soleil.

Of technological chemistry quite as much is introduced as is consistent with the character of the book. The student can get a general idea of the principal processes; the operator can learn the principles upon which his manipulations rest; the technical chemist of course must go elsewhere.

Formal chemistry, or the description of the elements, their compounds and reactions is carefully attended to, and in this respect the book is quite satisfactory. Of course it is impossible to describe every product and each reaction, so that the student who attempts to use this as a book of reference, may occasionally be disappointed, but of what work on chemistry cannot the same be said? There is a world of information on such topics as these scattered through the Scientific Journals, which ought to be collected; but who has the heart to undertake the immense toil?

Analytical chemistry has also received attention, and most of the recent processes in this department are described. They cannot teach analysis to one altogether unacquainted with it, but the student who has acquired some little experience in the laboratory will derive great assistance from these sketches of analytical methods.

*Transactions of the Illinois State Medical Society for the year* 1857. Chicago, 1857.

This is a neat octavo of 128 pages containing several very interesting papers. Among these we particularly notice a paper by Dr. Davis, on the *changes which take place in the blood in the continued form of fever,* and one by Dr. Chambers on *stomatitis materna* or the sore mouth of nursing women. The former of these would be far more valuable had Dr. Davis stated the method he employed in making the analysis of the blood. This is so intricate an examination and requires so many checks, that a full account of the process is absolutely necessary before the results can be received as established facts.

